# Supplementary figures and images for: Pulsatility Index as a Diagnostic Parameter of Reciprocating Wall Shear Stress Parameters in Physiological Pulsating Waveforms
Source: PLoS One. 2016 Nov 28;11(11):e0166426. doi: 10.1371/journal.pone.0166426 (PMC5125588; doi:10.1371/journal.pone.0166426)

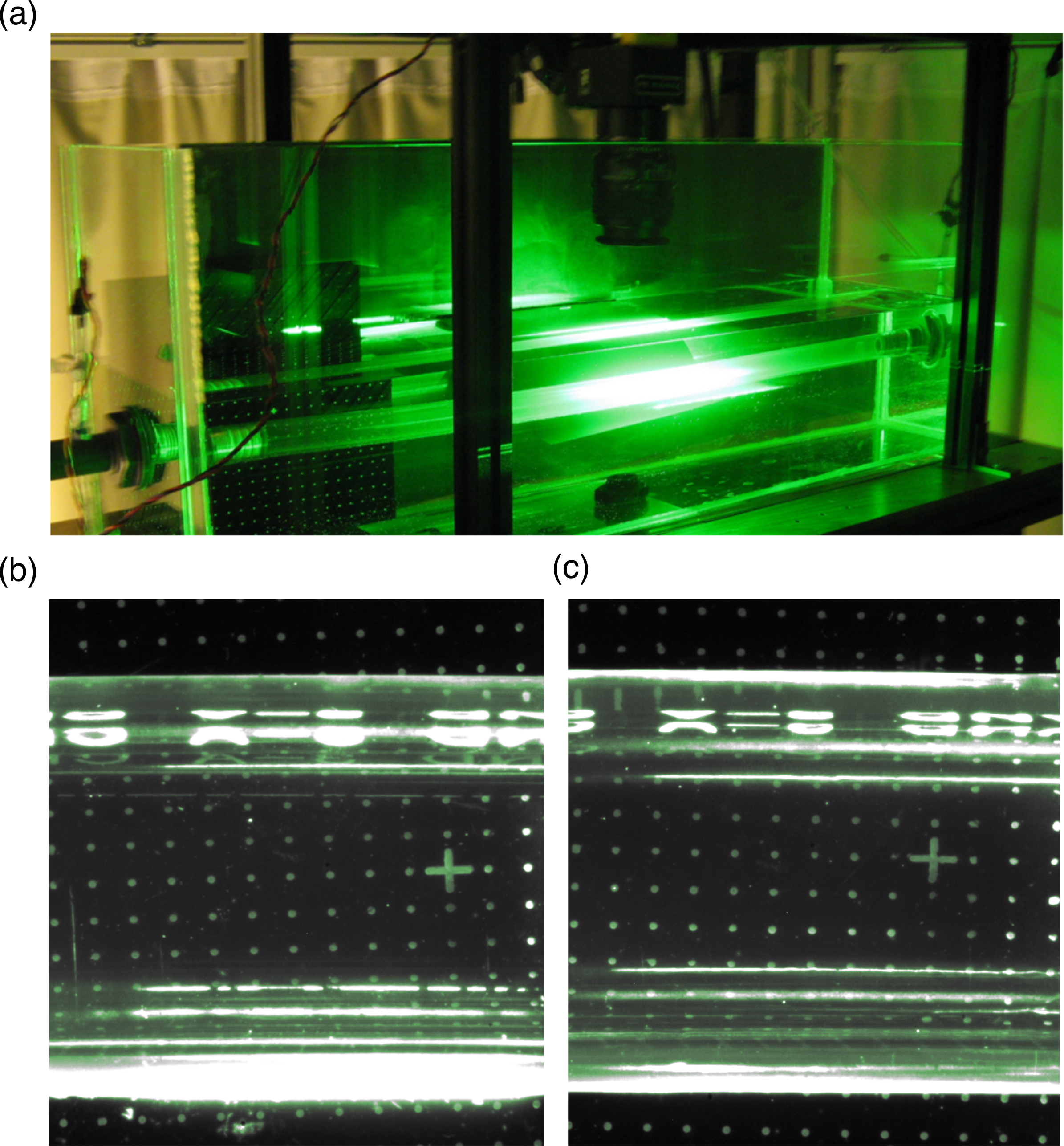

Supplement: S1 Fig — The aberrations are negligible due to very small differences in index of refraction (less than 0.001). (TIF) [file pone.0166426.s001.tif]
